# Plasma exo-hsa_circRNA_0056616: A potential biomarker for lymph node metastasis in lung adenocarcinoma

**DOI:** 10.7150/jca.30360

**Published:** 2020-04-06

**Authors:** Fei He, Xuejing Zhong, Zheng Lin, Jianbo Lin, Minglian Qiu, Xu Li, Zhijian Hu

**Affiliations:** 1 Department of Epidemiology and Health Statistics, School of Public Health, Fujian Medical University, Fuzhou, 350108, China; Fujian Provincial Key Laboratory of Tumor Microbiology, Fujian Medical University, Fuzhou, 350108, China; Key Laboratory of Ministry of Education for Gastrointestinal Cancer, Fujian Medical University, Fuzhou, 350108, China; 2 Department of Science and Education, The Affiliated Longyan First Hospital of Fujian Medical University, Longyan, 364000, China; 3 Department of Chest Surgery, The First Affiliated Hospital of Fujian Medical University, Fuzhou, 350001, China

**Keywords:** Lung adenocarcinoma, CXCR4, exosomes, circRNA, lymph node metastasis

## Abstract

**Background**: To investigate the relationship between CXCR4-related circular RNAs (circRNAs) in exosomes and lymph node metastasis of lung adenocarcinoma.

**Methods**: Totally 41 lung adenocarcinoma tissues (21 with lymph node metastasis and 20 without) were collected. Expression of CXCR4 protein was detected by western blotting analysis. A stable PC9/CXCR4-shRNA and PC14/CXCR4-shRNA knockdown lung adenocarcinoma cell lines were established and subjected to functional assays (cell proliferation, colony formation, migration and invasion) for phenotype changes. Exo-hsa-circRNAs (has-circRNAs in exosomes) were detected *in vivo* and in *vitro*. The diagnostic value of differentially expressed exo-has-circRNAs was evaluated.

**Results**: Expression levels of CXCR4 were higher in patients with lymph node metastasis than in those without (*P* = 0.001). Silencing *CXCR4* expression in PC9 and PC14 cell lines with short hairpin RNA could effectively abolish colony formation frequency, proliferation rate, migration rate, and the number of invasive cells (all *P* < 0.001). Exo_circRNA_0056616 was detected in both PC-9/CXCR4-shRNA cells and lung adenocarcinoma plasma at significantly higher levels than in the corresponding control (*P* < 0.001). When a receiver operating characteristic (ROC) curve for plasma exo-hsa_circRNA_0056616 levels and diagnosis of lymph node metastasis of lung adenocarcinoma was generated, a cutoff value of 0.394 was identified with an area under the curve of 0.812 (95% confidence interval 0.720-0.903), a sensitivity of 0.792, and specificity of 0.810.

**Conclusions**: Taken together, our findings suggested that CXCR4 was higher in the lung adenocarcinoma tissues with lymph node metastasis. Higher plasma levels of exo-hsa_circRNA_0056616 in these patients also suggest that this circRNA represents a potential biomarker for lymph node metastasis predictor in lung adenocarcinoma.

## Background

Lung cancer is one of the most frequent malignancies and leads cause of cancer-related deaths worldwide. The number of new cases of lung cancer is estimated to be 1.82 million (representing 12.9% of the total number of cancer cases diagnosed), while the number of deaths due to lung cancer is estimated to be 1.59 million (19.4% of all deaths due to cancer) 1. The high incident areas include China, which the number of new cases and deaths due to lung cancer being 73 million and 61 million, respectively, in 2015 2. 80 % of lung cancers are non-small cell lung cancer (NSCLC), with the primary pathology being lung adenocarcinoma. Due to the aggressive and metastatic phenotype of lung adenocarcinoma, the prognosis is extremely poor.

Tumor metastasis is a critical step in the malignant progression of tumors. Many studies have demonstrated that the chemokine receptor, CXCR4, is overexpressed in tumor tissues to regulate autocrine and paracrine signaling, angiogenesis, proliferation immune responses and other processes 3. It has been demonstrated that CXCR4 can bind to SDF-1, which activates ERK/AKT signaling or PI-3K signaling to regulate tumor cell proliferation 4. Taken together, these observations support the proposal that CXCR4 may be a useful biomarker for predicting tumor progression 5.

Exosomes secreted by the cells is a kind of extracellular vesicles with lipid bilayer structure, containing a variety of cell specific proteins, lipid, and nucleic acids, involved in intercellular communication, plays an important role in different physiological and pathological process 6. Exosomes can also carry non-coding RNAs (ncRNA) in the blood to achieve long-distance cell communication 6 and can promote microenvironments that facilitate the formation of lung cancer, as well as its invasive and metastatic phenotypes 7-10.

Circular RNAs (circRNAs) are a class of endogenous non-coding RNAs molecules with closed loops and high stability. Previously, circRNAs were considered miRNA sponges, and can bind to single or multiple miRNAs and regulate the expression of their downstream genes. However, a large number of studies have revealed the transcriptional and translational regulatory functions of circRNAs in various diseases, including cancers 11. CircRNAs are abundant and stable in exosomes and can continually play their roles after the exosomes are taken up by neighboring cells. When an expression profile of circRNAs was determined for exosomes obtained from serum samples from patients with lung cancer, this profile significantly differed from the profile obtained in parallel from healthy individuals 12. Thus, circRNAs may as biomarkers in lung cancer.

In this study, the aims were to: i) confirm whether expression of CXCR4 is associated with lymph node metastasis of lung adenocarcinoma *in vivo*; and ii) screen and identify circRNAs present in exosomes (exo-hsa_circRNA) that are associated with CXCR4 expression and have potential to serve as predictors of lymph node metastasis in lung adenocarcinoma.

## Methods

### Subjects and samples

A total of 90 lung adenocarcinomas samples, including 42 with lymph node metastasis and 48 without lymph node metastasis, were obtained from the Fujian Provincial Cancer Hospital (Fujian, China). Both groups of patients were diagnosed with lung cancer after pathological diagnosis, and the pathological type was adenocarcinoma. No chemotherapy or radiotherapy was performed before surgery; informed consent was signed; clinical data and tissue specimens were complete. Metastasis was determined based on postoperative pathological reports according to the following criteria: the number of lymph node dissection is ≥5, the number of metastasis is ≥1 for lymph node metastasis; the number of lymph node dissection is ≥5, and the number of metastasis=0 is no lymph node metastasis. Exclusion of severe cardiac and brain disease, secondary adenocarcinoma and recurrence of lung adenocarcinoma.

Peripheral venous blood (5 mL) was collected from each patient prior to surgery into EDTA anticoagulant tubes. After a centrifugation step at 2000 rpm for 10 min, the samples were transferred to RNase-free centrifuge tubes and stored at -80 °C. Meanwhile, 41 tumor tissue specimens were immediately preserved in liquid nitrogen after their resection and were stored at -80 °C until analyzed.

Information regarding patients and their samples were obtained with permission from the Institutional Medical Ethics Committee of Fujian Medical University. Written informed consent forms were obtained from all the participating subjects.

### Cell culture

PC9 cells and PC14 cells were obtained from YUBO Biological Company (Shanghai, China) and 293T cells were obtained from ATCC (Shanghai, China). Cell lines were maintained in Dulbecco's Modified Eagle Medium (DMEM, Gibco, NY, USA) supplemented with 10% fetal bovine serum (FBS, Gibco) and 1% penicillin-streptomycin (Gibco) at 37 °C in a 5% CO_2_ humidified incubator.

### Western blotting analysis

Cells were lysed in whole-cell lysate buffer (Solarbio, Beijing, China) and then resolved by 10% or 12% sodium dodecyl sulfate-polyacrylamide gel electrophoresis (SDS-PAGE). The separated proteins were then transferred to polyvinylidene difluoride (PVDF) membranes (Pall, NY, USA) and blocked with 5 % skim milk. The membranes were incubated with primary antibodies overnight at 4 °C and with appropriate secondary antibodies at room temperature for 1 h. The primary antibodies recognized CXCR4 and actin (Abcam, MA, USA), glyceraldehyde 3-phosphate dehydrogenase (GAPDH, Santa Cruz Biotechnology, TX, USA), CD81 and CD63 (System Biosciences, CA, USA). Protein bands were visualized with reagents from an enhanced chemiluminescence kit (Pierce, IL, USA) and band intensities were analyzed with ImageJ software (NIH, MD, USA). Detection of actin was performed as a loading control.

### Generation and characterization of a stable cell line expressing low levels of CXCR4

Short hairpin RNAs (shRNAs) targeting *CXCR4* were designed with online RNAI design software (http://www.protocol-online.org/prot/Research_Tools/Online_Tools/SiRNA_Design/) and synthesized by Shanghai Sangon Ltd. (Shanghai, China). These shRNAs were each cloned into the PLVX-shRNA-PURO lentivirus vector (Takara Biomedical Technology, Beijing, China) to generate fusion proteins with ZsGreen fluorescent protein (Takara Biomedical Technology). The corresponding plasmids were then transfected into 293T cells with Xfect Transfection reagent (Takara Biomedical Technology), according to the manufacturer's directions. Puromycin selection and screening of the transfected cells based on GFP expression resulted in the identification of cells to produce lentivirus. Infections of the PC9 and PC14 cells were conducted with an Endo-free Plasmid Maxi Kit (OMEGA, Norcross, GA) and Lenti-X™ Lentiviral Expression Systems (Takara Biomedical Technology, Beijing, China), according to the manufacturers' protocols. The infected cells were named PC9/CXCR4-shRNA and PC9/shCtrl cells, PC14/CXCR4-shRNA and PC14/shCtrl cells, respectively. GFP expressions in these two sets of infected cells were confirmed with a fluorescence inverted microscope and the infected cells were subsequently cultured as single cell colonies. After expressions of the appropriate shRNAs were confirmed, these cell lines were used for experimentation.

### Soft agar colony assay

PC9/CXCR4-shRNA, PC9/shCtrl, and PC9, PC14/CXCR4-shRNA, PC14/shCtrl, and PC14 cells were digested and counted to adjust the cell density of each to 1 × 10^3^ cells/mL. Approximately 500 cells (0.5 ml) of each cell suspension were then added to volumes of 9.4 ml DMEM. Twenty-four well plates containing 5% agar at 37 °C had 40 cells added per well, with triplicate wells prepared in duplicate for each sample. Clone formation rates (%) were calculated as follows: [number of clones observed × 5 / number of inoculated cells] x 100%.

### CCK8 assay

For the CCK8 assays, PC9/CXCR4-shRNA, PC9/shCtrl, PC9, and PC14/CXCR4-shRNA, PC14/shCtrl, and PC14 cells were plated in 96-well plates (4 × 10^3^ cells/well). After 24 h at 37 °C and 5% CO_2_, 10 mL of CCK8 buffer was added to each well. After 1-2 h, optical density values were measured at 450 nm with a Multiskan^™^FC microplate photometer (ThermoFisher Scientific, MA, USA). The cell viability values determined for each group were subsequently normalized to the cell viability value of the untreated control cells. This assay was repeated at least three times.

### Wound healing assay

PC9/CXCR4-shRNA, PC9/shCtrl, PC9, and and PC14/CXCR4-shRNA, PC14/shCtrl, and PC14 cells suspension were adjusted to 3 × 10^6^ cells/mL. Then, 200 μL of each cell suspension was added to 2 mL of complete medium in 6-well plates. When the cells reached 80% confluency, a pipette was used to draw a vertical line in each of the wells. The scratched wells were incubated for 48 h and then each well was imaged to evaluate cell migration.

### Transwell assay

For the transwell assays, the bottom membrane of each well (12 mm, Corning, MA, USA) was coated with 50 mg/L matrigel (diluted 1:8 in DMEM) then air dried at 4 °C. PC9/CXCR4-shRNA, PC9/shCtrl, PC9, and PC14/CXCR4-shRNA, PC14/shCtrl, and PC14 cells suspension densities were adjusted to 5 × 10^5^ cells/mL, and 100 μL of each cell suspension was added to the top of each Transwell chamber. One mL of DMEM/10% FBS was added to the lower chamber. After 24 h, cotton swabs were used to wipe each upper membrane and then the Transwells were placed in 70% alcohol to fix the cells on the lower membrane. Subsequently, these cells were stained with 100 μL Giemsa stain for 5 min before the membranes were imaged and the stained cells were counted.

### Analysis of plasma exosomes

Plasma exosomes were extracted from lung adenocarcinoma samples and PC9/CXCR4-shRNA, PC9/shCtrl, and PC9 cells by using an ExoQuick Isolation kit (System Biosciences, USA).

Total RNA was subsequently extracted from these samples with a QIAGEN RNeasy Mini kit (Qiagen, Duesseldorf, Germany), according to the manufacturer's instructions. Real-time quantitative reverse transcription-polymerase chain reactions (RT-qPCR) were performed with SYBR® Premix Ex Taq^TM^ II (Tli RNaseH Plus, TaKaRa, Dalian, China), according to the manufacturer's instructions. Divergent primers for exo-hsa_circRNA_0056616 and primers for *CXCR4* and *GAPDH,* which was to be the reference gene, were designed by Biocan (Shenzhen, China) and synthesized by TaKaRa. The normalization of qPCR in the expression of exo-hsa circRNA 0056616 was referring to Wang's methods 13. All RT-qPCR primer sets are listed in Table S1 and S2.

### Statistical analysis

Statistical analysis was performed with SPSS 22.0 (IBM, NY, USA). Differences in the values for exo-hsa_circRNA_0056616 and CXCR4 between groups were determined by using the t-test and analysis of variance (ANOVA). Wilcoxon rank sum test and Pearson correlation analysis were used to determine correlations between expression levels of exo-hsa_circRNA_0056616 and CXCR4 and clinical indexes. A receiver operating characteristics (ROC) curve was generated for exo-hsa_circRNA_0056616 for lymph node metastasis detection. A *P* value less than 0.05 was considered statistically significant.

## Results

### CXCR4 was upregulated and associated with lymph node metastasis in lung adenocarcinoma

Expression of CXCR4 in 41 lung adenocarcinoma tissues (21 lymph node metastasis tissues and 20 without) was detected by western blotting analysis, Actin was used as a loading control Fig. S1A). Results showed that the median expression value of CXCR4 was 0.576 (interquartile range, 0.371-0.757) in 41 lung adenocarcinoma tissues. The median levels of CXCR4 expression was upregulated in metastasis tissues 0.624 (interquartile range: 0.564-0.998) than in those of without metastasis tissues 0.432 (interquartile range: 0.294-0.577) (Z = -3.417, *P* = 0.001) (Fig. S1B). Together, these results suggest that overexpression of CXCR4 in lung adenocarcinoma is related to lymph node metastasis.

### Establishment of stable CXCR4 knockdown cell lines

To determine whether shRNAs affects the expression of CXCR4 in a lung adenocarcinoma cell line, three shRNAs (shRNA-1, shRNA-2, and shRNA-3) specifically targeting *CXCR4* were generated with lentivirus system, respectively. As shown in Figure S2A, electrophoresis results showed that shRNA clones 3-8, and a representative Sanger sequencing read for one of those clones is shown in Figure S2B. three shRNAs specifically targeting *CXCR4* were transfected into PC9 cell line, as well as a scrambled control shRNA (shRNA-c), respectively. In RT-qPCR assays revealed that three shRNAs could specifically down-regulate the levels of *CXCR4* compared with the controls (*P* < 0.05) (Fig. S2C). However, shRNA1, subsequently referred to as CXCR4-shRNA1 (or PC9-KD in the figures) was selected to perform subsequent experiments. The CXCR4-shRNA1, PC9/shCtrl (or PC9-C in the figures) and untransfected PC9 cells were detected with microscopy and fluorescence microscopy (Fig. S2D). The silencing effect was checked by western blotting and RT-qPCR analysis, and results showed that shRNA1 could specifically down-regulate expression of CXCR4 (Fig. S2E and S2F).

The levels of CXCR4 protein detected in the PC9/CXCR4-shRNA1 cells versus the PC-9/shCtrl cells were 0.239 ± 0.016 versus 1.016 ± 0.020, respectively (*P* < 0.001) (Fig. S2E). In the RT-qPCR assays, the relative expression levels of *CXCR4* mRNA were 0.288 ± 0.013 for the PC9/CXCR4-shRNA1 cells, and 1.047 ± 0.056 and 1.001 ± 0.055 for the PC9/shCtrl and un-transfected PC9 cells, respectively (*P* < 0.001) (Fig. S2F). Therefore, a significant 3.6-fold decrease in *CXCR4* mRNA levels was achieved by using CXCR4-shRNA1 to target *CXCR4* (*P* < 0.001).

### Knockdown of CXCR4 abolishes a typical metastasis phenotype

**Figure 1:**
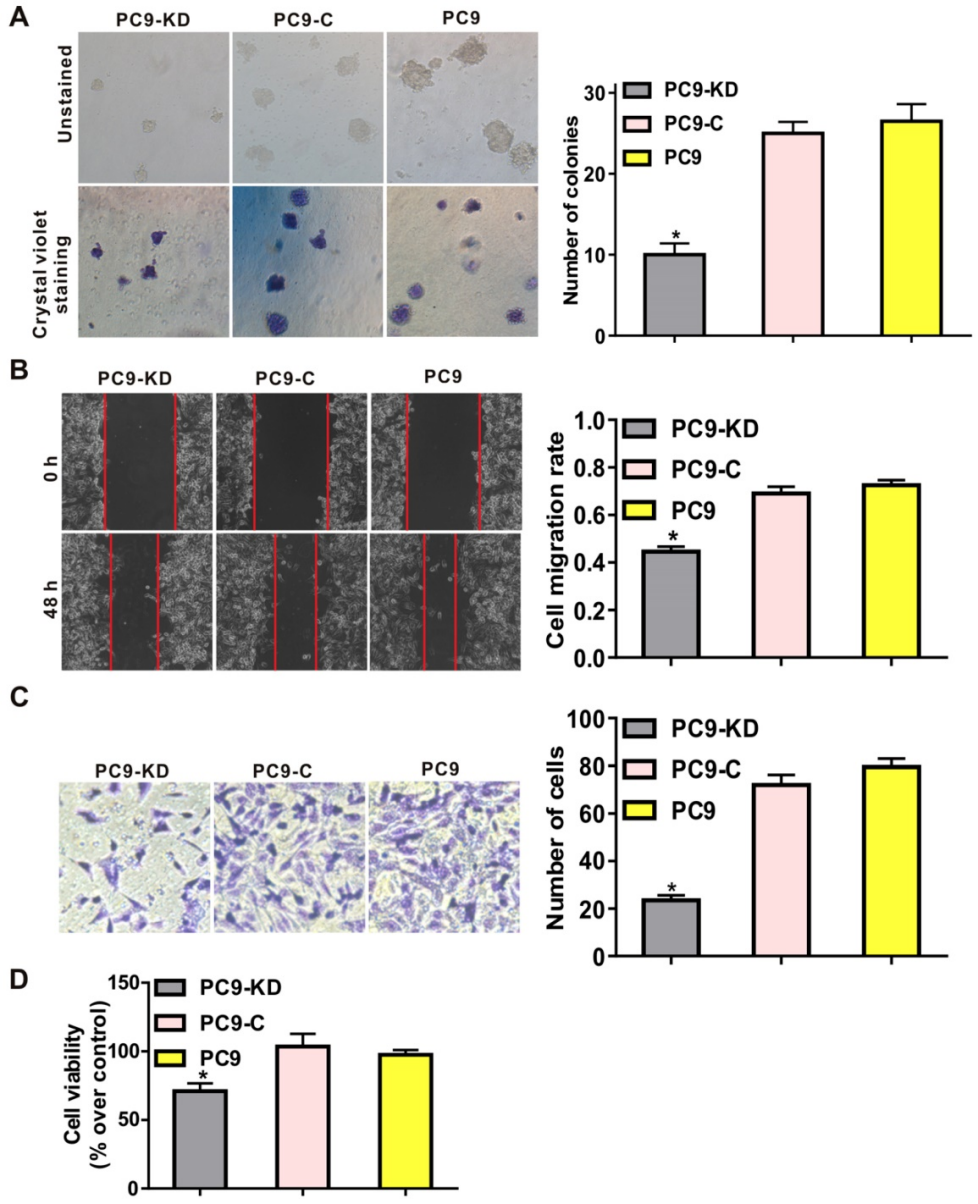
Effect of *CXCR4* silencing on various phenotypes of the PC9 cell line. Low levels of CXCR4 expression were compared with higher levels of CXCR4 expression in: (A) soft agar colony formation assays, (B) wound healing assays, (C) transwell assays, and (D) CCK8 assays. These assays were performed to evaluate colony formation, cell migration, cell invasion and cell viability, for the PC9 cell groups indicated, respectively. In panel C, crystal violet stained cells (at left) and the corresponding quantitation of these stained cells (at right) indicate the number of invasion cells for each group. PC9-KD: PC9/CXCR4-shRNA1 group; PC9-C: PC9/shCtrl group; PC9: untransfected PC9 group.

To characterize the function of CXCR4 silencing in a lung adenocarcinoma cell line, functional assays revealed that CXCR4 silencing could effectively inhibit the tumorigenic phenotype by reducing frequencies of colony formation in soft agar compared with cells treated with scramble shRNAs (*P* < 0.001) (Fig. 1A and Fig. S3A). To investigate the effects of CXCR4 silencing on tumor metastasis, both migration and invasion assays showed that migratory and invasive abilities were significantly decreased in PC9/CXCR4-shRNA1 and PC14/CXCR4-shRNA1 cells compared to the control cells (*P* < 0.001) (Fig. 1B and C; Fig. S3B and C). To evaluate cell proliferation, CCK8 assay demonstrates that CXCR4 silencing could decrease cell viability compared to the control cells (*P* < 0.01) (Fig. 1D). Taken together, all these results indicate that *CXCR4* silencing *regulates* the hallmarks of the metastasis process by inhibiting colony formation, cell proliferation, migration and invasive in lung adenocarcinoma cell line.

### CXCR4-regulated PC9 phenotype changes associated with hsa_circRNA_0056616

**Figure 2:**
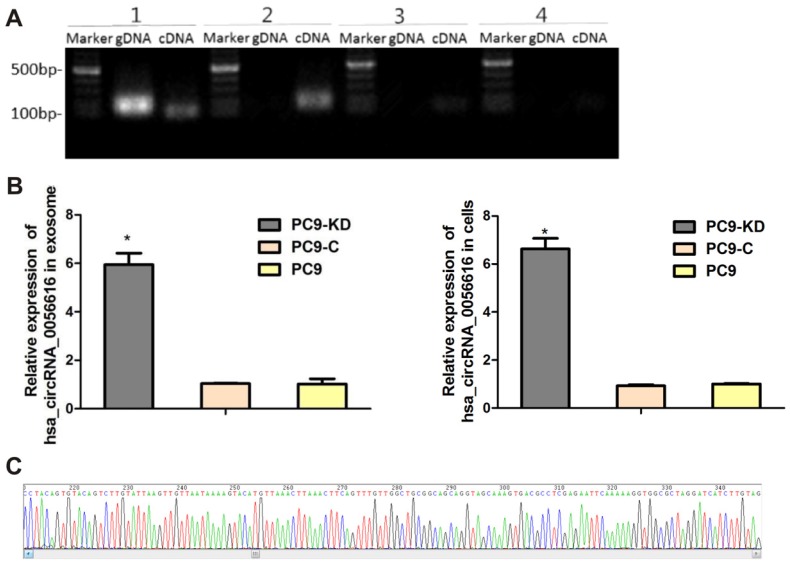
Identification and detection of hsa_circRNA_0056616 in lung adenocarcinoma cells and exosomes. (A) Based on a literature search, three circRNAs were previously reported to be related to CXCR4. These were detected in genomic DNA (gDNA) and complementary DNA (cDNA) samples by PCR. [1: GAPDH (as a control); 2: hsa_circRNA_0056616; 3: hsa_circRNA_0117403; 4: hsa_circRNA_0056615]. Marker: DNA ladder. (B) Hsa_circRNA_0056616 was detected by PCR in exosomes and cells from the cell groups indicated. *P < 0.001. PC9-KD: PC9/CXCR4-shRNA1 group; PC9-C: PC9/shCtrl group; PC9: blank control group. (C) Sequencing of hsa_circRNA_0056615.

A search was performed of the circbase, circRNABase, and Circ2Traits databases to identify potential circRNAs that may be associated with CXCR4. Hsa_circRNA_0056615, hsa_circRNA_0056616, and hsa_circRNA_0117403 were identified (Table S3. RT-qPCR was performed to detect these circRNAs, as shown in Fig. 2A, the only of hsa_circRNA_0056616 was detected in the cells and exosomes of the PC9/CXCR4-shRNA1 cells. And the sequence of amplified hsa_circRNA_0056616 DNA was verified by sanger sequencing (Fig. 2B). Futhermore, the expression levels of hsa_circRNA_0056616 were detected in exosomes that were isolated from three PC9 groups by RT-qPCR assays, our data have demonstrated that the levels of hsa_circRNA_0056616 were found to be 5.940 ± 0.479 for the PC-9/CXCR4-shRNA1 groups, and 1.034 ± 0.016 and 1.015 ± 0.219 for the negative control PC-9/shCtrl and blank control PC9 groups, respectively (*P* < 0.001) (Fig. 2C, left panel). Meanwhile, the mean expression level of hsa_circRNA_0056616 in the PC9/CXCR4-shRNA1 cells was 6.631 ± 0.439, compared with levels of 0.931 ± 0.045 and 1.000 ± 0.030 in the negative control PC9/shCtrl and blank control PC9 cells, respectively (*P* < 0.001) (Fig. 2C, right panel). Thus, our findings indicate that the hsa_circRNA_0056616 is remarkably upregulated in PC9/CXCR4-shRNA1 cells and PC9/CXCR4-shRNA1 cells-derived exosomes.

### Exo-hsa_circRNA_0056616 as a potential marker of lymph node metastasis of lung adenocarcinoma

For this study, 42 patients with lung adenocarcinoma metastasis, including 19 male patients and 23 female patients (mean age, 57.71 ± 8.41 y) were enrolled. In addition, 48 patients with non-metastatic lung adenocarcinoma, including 25 males and 23 females (mean age, 59.60 ± 7.47 y) were enrolled. The gender proportions and mean ages for the two groups have no significant difference (Table 1).

**Figure 3:**
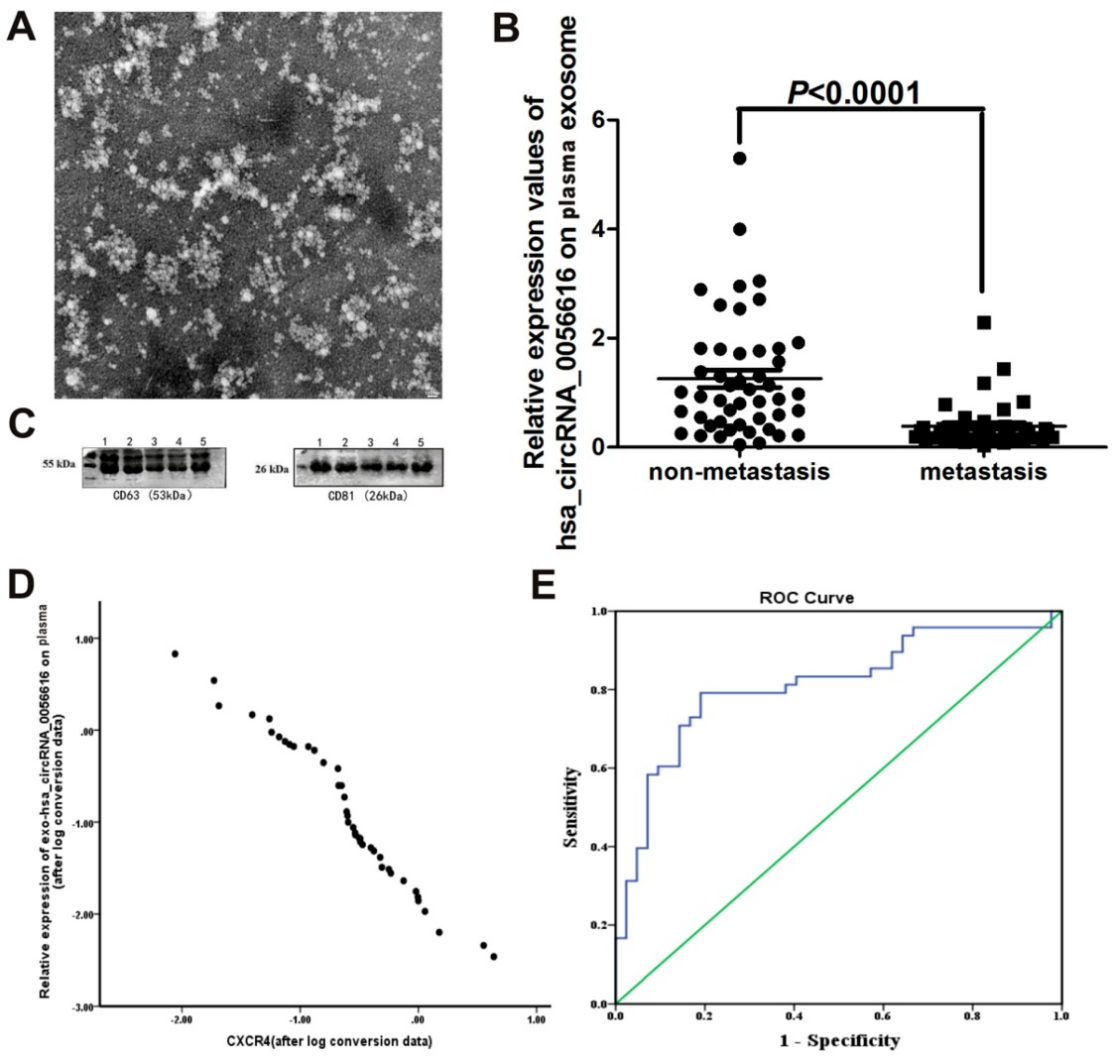
Exo-hsa_circRNA_0056616 as a potential marker of lymph node metastasis of lung adenocarcinoma. (A) Plasma exosomes of lung adenocarcinoma patients were examined with transmission electron microscopy. Rounded and oval-shaped vesicles exhibiting obvious heterogeneity were observed. The diameters of these cells ranged from 40-100 nm. Scale bar: 100 nm. (B) Expression levels of exo-hsa_circRNA_0056616 in 90 plasma samples according to the metastasis status of the samples (meam±standard deviation). (C) A representative western blot of the detection of CD63 and CD81 in plasma samples from a lung adenocarcinoma patient with metastasis. (D) Plasma levels of exo-hsa_circRNA_0056616 expression was plotted in relation to levels of CXCR4 detected in the corresponding samples. (E) The ROC curve (in blue) generated from the detection of exo-has_circRNA_0056616 in 90 plasma samples in relation to the diagnosis of lymph node metastasis of lung adenocarcinoma for the samples. The green line represents non predictive values.

We examined exosomes morphology in plasma using transmission electron microscopy (Fig. 3A). Our results showed a rounded and oval-shaped vesicle with diameters ranging from 40-100 nm exhibited obvious heterogeneity. Furthermore, as shown in Figure 3C, western blotting analysis for exosomes marker proteins CD63 and CD81 were verified in plasma sample collected from a lung adenocarcinoma patient with metastasis.

When expression of exo-hsa_circRNA_0056616 was detected in plasma samples collected from this cohort of lung adenocarcinoma patients, the patients with lymph node metastasis had significantly lower levels of exo-hsa_circRNA_0056616 than the patients without lymph node metastasis (Z = -5.079, *P* = 0.001) (Fig. 3B). Moreover, expression of exo-hsa_circRNA_0056616 was found to negatively correlate with CXCR4 expression (*r* = -0.979, P < 0.05) (Fig. 3D). Furthermore, higher expression levels of exo-hsa_circRNA_0056616 in plasma correlated with higher protein levels of CXCR4 in lung adenocarcinoma tissue.

Levels of exo-hsa_circRNA_0056616 in plasma were further examined in relation to T1/T2 lung adenocarcinoma versus T3/T4 lung adenocarcinoma. The T1/T2 stage patients had higher plasma levels of exo-hsa_circRNA_0056616 than the T3/T4 stage patients, and the difference was statistically significant (P = 0.002) (Table 1). In addition, the patients classified with lung adenocarcinoma M0 or lung adenocarcinoma I-II had higher levels of plasma exo-hsa_circRNA_0056616 than the patients with lung adenocarcinoma M1 (*P* = 0.004) or lung adenocarcinoma III-IV (*P* = 0.001), respectively (Table 1). In contrast, plasma levels of exo-hsa_circRNA_0056616 did not correlate with the age, gender, tumor size, or anatomical classification of lung adenocarcinoma in the patients examined (Table 1). In addition, a significant negative correlation was observed between the expression of exo-hsa_circRNA_0056616 and T stage, M stage, and TNM grade of lung adenocarcinoma (*P* < 0.05) (Table 2). Moreover, there was no significant correlation between tumor size and anatomical classification (*P* > 0.05) (Table 2).

When a ROC curve for plasma exo-hsa_circRNA_0056616 levels and diagnosis of lymph node metastasis of lung adenocarcinoma was generated (n = 90) (Fig. 3E), the area under curve (AUC) was 0.812 (standard error: 0.047; 95% confidence interval, 0.720, 0.903), the maximum Jordan index value was 0.602, and the sensitivity and specificity were 0.792 and 0.810, respectively. As a result, a cutoff value of 0.394 was identified.

## Discussion

It was previously reported that high levels of CXCR4 are expressed in lung adenocarcinoma 14, 15. In addition, high levels of CXCR4 have been closely related to lymph node metastasis, a high T stage, and patient prognosis 16. In the present study, our data demonstrate that CXCR4 expression is associated with lymph node metastasis of lung adenocarcinoma in the cohort examined. In addition, hsa_circRNA_0056616 was found to be present at higher levels in PC9 lung adenocarcinoma cells and their exosomes following the silencing of *CXCR4* with shRNA. Detection of hsa_circRNA_0056616 in tumor tissues and plasma also distinguished lung adenocarcinoma patients with or without lymph node metastasis (cutoff value, 0.394). Thus, plasma levels of exo-hsa_circRNA_0056616 may represent a valuable biomarker for theranostics of lymph node metastasis lung adenocarcinoma.

The initial observation in the present study that CXCR4 was highly expressed in carcinoma tissues of lung adenocarcinoma metastasis patients is consistent with the hypothesis that CXCR4 plays an important role in many solid tumor metastases 17. For example, increased expression of *CXCR4* has been associated with accelerated tumor progression and tumor cell proliferation 18. Similarly, our data demonstrate that silencing *CXCR4* expression in PC9 and PC14 cell lines could effectively abolish cell proliferation. These results are also consistent with previous results 19, 20. It has also been reported that interactions between CXCR4 and SDF-1 lead to activation of G protein-mediated signaling pathways and downregulation of RAS and PI3 kinase to affect cell motility 21, 22. Furthermore, CXCR4/SDF-1 complexes have been shown to activate protein kinase B, mitogen-activated protein kinases, and nuclear factor kappa B in combination with phosphatidylinositol 3-kinases or Src family kinases and other downstream signaling proteins to promote the expression of heparanase and metalloproteinase-9 and degradation of extracellular matrix 23. As a result, tumor cells can gain access to adjacent tissues as part of tumor invasion and metastasis processes 23. In the present study, our data showed that *CXCR4* silencing regulates the hallmarks of the metastasis process by inhibiting colony formation, cell proliferation, migration and invasive in PC9 and PC14 cell lines.

Currently, increasing evidence has revealed that cancer-related microRNAs (miRNAs) and regulation of circRNA-miRNA interactions in cancer signaling pathways are active. It has been demonstrated that circRNAs can act as stable endogenous RNAs, competitive endogenous RNAs, or miRNA sponges to regulate the expression of their downstream gene 24. Similarly, when circRNAs contain multiple tandem binding sites for miRNAs they can regulate miRNA activity by acting like an miRNA sponge 25. For example, the circRNA, CiRS-7, acts as a sponge of miR-7 since it contains more than 70 miR-7 conserved binding sites, and this allows CiRS-7 to negatively regulate miR-7 26. Moreover, Chou 27 reported that overexpression of miR-7 is associated with poor prognosis in lung cancer, while inhibition of miR-7 expression may decrease cancer cell proliferation and increase apoptosis 28. CircRNAs that contain multiple MREs are more effective than traditional miRNA inhibitors, thereby providing an alternative to linear sponges. In malignant melanoma cell lines, a circRNA that acted similar to an miRNA sponge produced a longer lasting effect that resulted in a significant anti-cancer effect 29. CircRNAs have a closed loop structure that makes them less accessible to nucleases and more stable than linear RNAs. This characteristic has obvious advantages for the development and application of new markers. Thus, circRNAs may be the best miRNA inhibitors identified to date, and it is anticipated that they will be valuable targets in the diagnosis and treatment of tumors 30, 31.

To date, the potential for circRNAs to serve as tumor biomarkers has been evaluated in tumor tissues and cell lines 32-35. It remains for the clinical potential of circRNAs to be established. However, it has been observed by Li et al. 12 that the expression of circRNAs in exosomes was higher than in normal cells. Levels of circRNAs in serum exosomes of colon cancer patients were higher than in healthy individuals, and the expression levels in the exosomes correlated with the expression levels in cells (R = 0.43) [15]. The results of the present study regarding the detection of circRNAs in exosomes isolated from the peripheral blood of patients with lung adenocarcinoma are consistent with these results. In particular, exo-hsa_circRNA_0056616 was identified as a potential biomarker for lymph node metastasis. Furthermore, expression of exo-hsa_circRNA_0056616 was found to correlate with T, N, and M stages, as well as TNM grade, in patients with lung adenocarcinoma. With an area under the curve value of 0.812, this biomarker exhibited high accuracy and reliability for detecting lung adenocarcinoma lymph node metastasis versus non-metastatic lung adenocarcinoma, and this is an encouraging finding that needs to be confirmed in future studies.

There were limitations associated with the present study. First, due to the focus of this study on metastases, the PC-9 cell line was chosen. However, additional studies are needed to address whether the results obtained with PC-9 cells can be confirmed in other cell lines. Second, circRNAs contain multiple MREs that are more effective than traditional miRNA inhibitors and they can potentially serve as an alternative to linear sponges. A correlation between circRNA expression and mRNA expression was analyzed in relation to CXCR4, although, the miRNA and mRNA targets of hsa_circRNA_0056616 were not validated. Moreover, although this study quantitatively analyzed the expression of exo-hsa_circRNA_0056616 in plasma samples obtained from lung adenocarcinoma patients, a positioning analysis was not conducted.

## Conclusions

In summary, the present study demonstrated that CXCR4 was upregulated and associated with lymph node metastasis, and exo-hsa_circRNA_0056616 in plasma to serve as a potential biomarker for theragnostic of lymph node metastasis in lung adenocarcinoma. However, it remains for these results to be validated in a larger number of samples, and further studies are warranted to investigate the molecular details that are responsible for the regulation and actions of hsa_circRNA_0056616. Nevertheless, the present findings are of value and they advance our understanding of lung adenocarcinoma metastasis.

## Supplementary Material

Supplementary figures and tables.

## Figures and Tables

**Table 1:** Plasma levels of exo-hsa_circRNA_0056616 and clinicopathological features of the cohort examined.

Clinicopathological features	n	Exo-hsa_circRNA_0056616	
		M (P_25_, P_75_)	*P*-value
*Gender*			
Male	44	0.569 (0.254, 1.102)	0.765
Female	46	0.370 (0.218, 1.237)	
*Age*			
< 60.5 y	45	0.467 (0.168, 1.344)	0.869
≥ 60.5 y	45	0.394 (0.274, 0.846)	
*T stage*			
T1 / T2	61	0.658 (0.309, 1.476)	**0.002**
T3 / T4	27	0.282 (0.156, 0.547)	
*M stage*			
M0	65	0.658 (0.306, 1.344)	**0.004**
M1	25	0.288 (0.165, 0.515)	
*TNM classification*			
I-II	50	0.830 (0.325, 1.730)	**0.001**
III-IV	37	0.287 (0.164, 0.380)	
Tumor size^*^			
(Maximum diameter of tumor)	33	0.884 (0.423, 1.476)	0.122
< 2.40 cm	33	0.347 (0.283, 1.243)	
*Anatomical classification*			
Central		0.438 (0.161, 1.132)	0.205
Surrounding		0.672 (0.309, 1.385)	

M: median; P_25_: 1st quartile (Q1); P_75_: 3rd quartile (Q3).

**Table 2:** Correlations between plasma levels of exo-hsa_circRNA_0056616 and lung adenocarcinoma features.

Clinicopathological features	Plasma hsa_circRNA_0056616	
	r_s_	*P*-value
T stage	-0.318	**0.003**
M stage	-0.308	**0.003**
TNM classification	-0.466	**0.001**
Tumor size	-0.232	0.060
Anatomical classification	0.154	0.207

## Abbreviations

circRNAs: circular RNAs
exo-hsa-circRNAs: has-circRNAs in exosomes
SDS-PAGE: sodium dodecyl sulfate-polyacrylamide gel electrophoresis
PVDF: polyvinylidene difluoride
GAPDH: glyceraldehyde 3-phosphate dehydrogenase
shRNAs: short hairpin RNAs
ROC: receiver operating characteristics
AUC: area under curve
MREs: miRNA response elements
