# Supplementary material for: Plasma exo-hsa_circRNA_0056616: A potential biomarker for lymph node metastasis in lung adenocarcinoma
Source: J Cancer. 2020 Apr 6;11(14):4037–46. doi: 10.7150/jca.30360 (PMC7196257; doi:10.7150/jca.30360)
Supplement: Supplementary file 1 — Supplementary figures and tables. [file jcav11p4037s1.pdf]

Figure S1. Expression levels of CXCR4 detected in lung adenocarcinoma tissues. (A) Western blot analysis was used to detect expression levels of CXCR4. Detection of actin served as a loading control. The sample number labels shown in black and red represent samples from patients with and without lymph node metastasis, respectively. (B) Quantitation of CXCR4 levels (median, [P_{25}-P_{75}]) according to metastasis.

Figure S2. Validation of a PC-9 stable cell line expressing low levels of CXCR4. (A) PCR amplification of CXCR4-shRNA cassettes in: (1) negative control (ddH_2O); (2) DNA marker; and (3-8) CXCR4-shRNA clones 3-8 samples. (B) Sequencing of a representative PCR product from A. (C) Detection of *CXCR4* mRNA in the PC-9/CXCR4 stable cell line with and without shRNA silencing as indicated (*P < 0.01). (D) Cell morphology (magnification, 100×) was observed by microscope and confocal microscope. GFP imaging was performed to confirm expression of the infecting lentiviruses. (E) Western blotting was performed to detect expression of CXCR4 in the cell lines indicated. Detection of GAPDH was included as a loading control. (F) Expression of *CXCR4* mRNA was detected in RT-qPCR assays of the indicated cell groups. PC9-KD: PC-9/CXCR4-shRNA1 group; PC9-C: PC-9/shCtrl group; PC9: untransfected PC-9 group. * $P < 0.001$.

Figure S3. Effect of *CXCR4* silencing on various phenotypes of the PC14 cell line. Low levels of CXCR4 expression were compared with higher levels of CXCR4

expression in: (A) soft agar colony formation assays, (B) wound healing assays, and (C) transwell assays. These assays were performed to evaluate colony formation, cell migration, and cell invasion，for the PC14 cell groups indicated, respectively. In panel C, crystal violet stained cells (at left) and the corresponding quantitation of these stained cells (at right) indicate the number of invasion cells for each group. PC14-KD: PC14/CXCR4-shRNA1 group; PC14-C: PC14/shCtrl group; PC14: untransfected PC14 group.

Table S1. ShRNA sequences for targeting *CXCR4*

Table S2. Sequences of the primers used in RT-qPCR assays.

Table S3. CXCR4-related circRNAs identified in database search

Figure S1

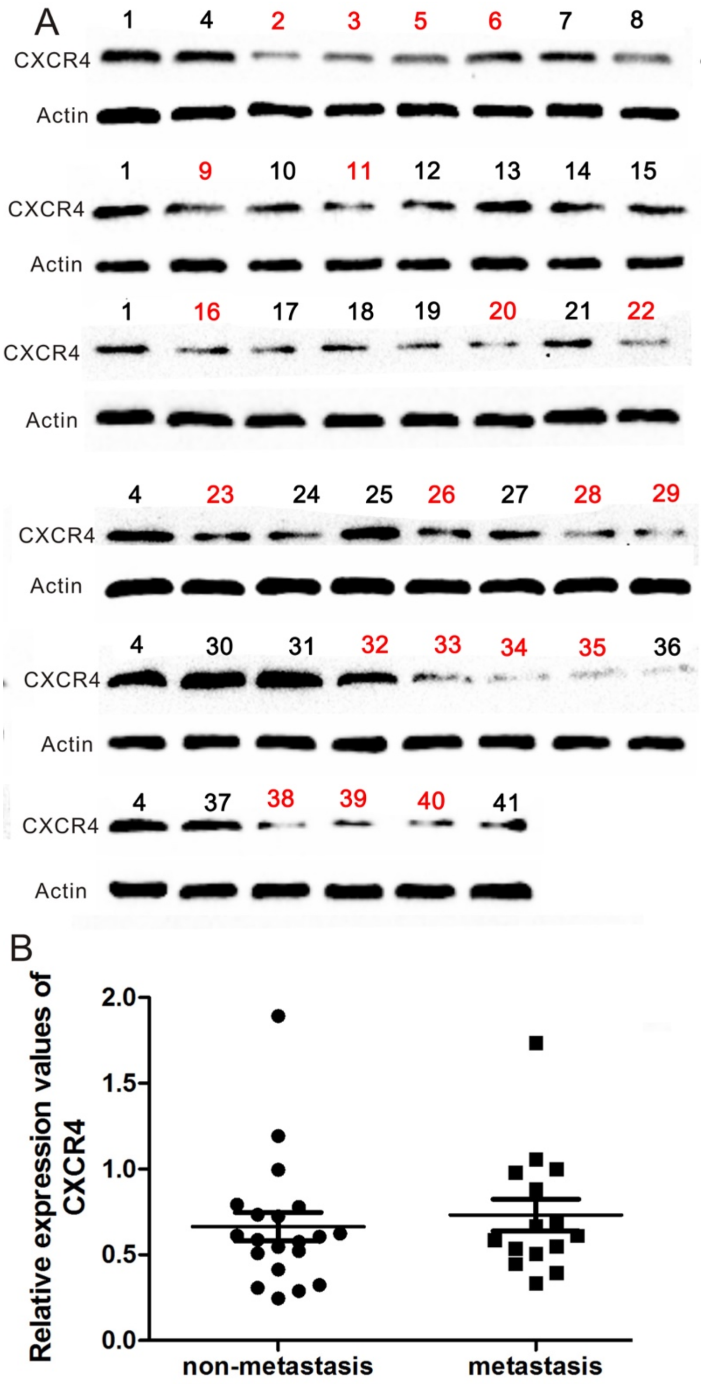
A
1 4 2 3 5 6 7 8
CXCR4
Actin
1 9 10 11 12 13 14 15
CXCR4
Actin
1 16 17 18 19 20 21 22
CXCR4
Actin
4 23 24 25 26 27 28 29
CXCR4
Actin
4 30 31 32 33 34 35 36
CXCR4
Actin
4 37 38 39 40 41
CXCR4
Actin
B
Relative expression values of CXCR4
2.0
1.5
1.0
0.5
0.0
non-metastasis
metastasis

Figure S2

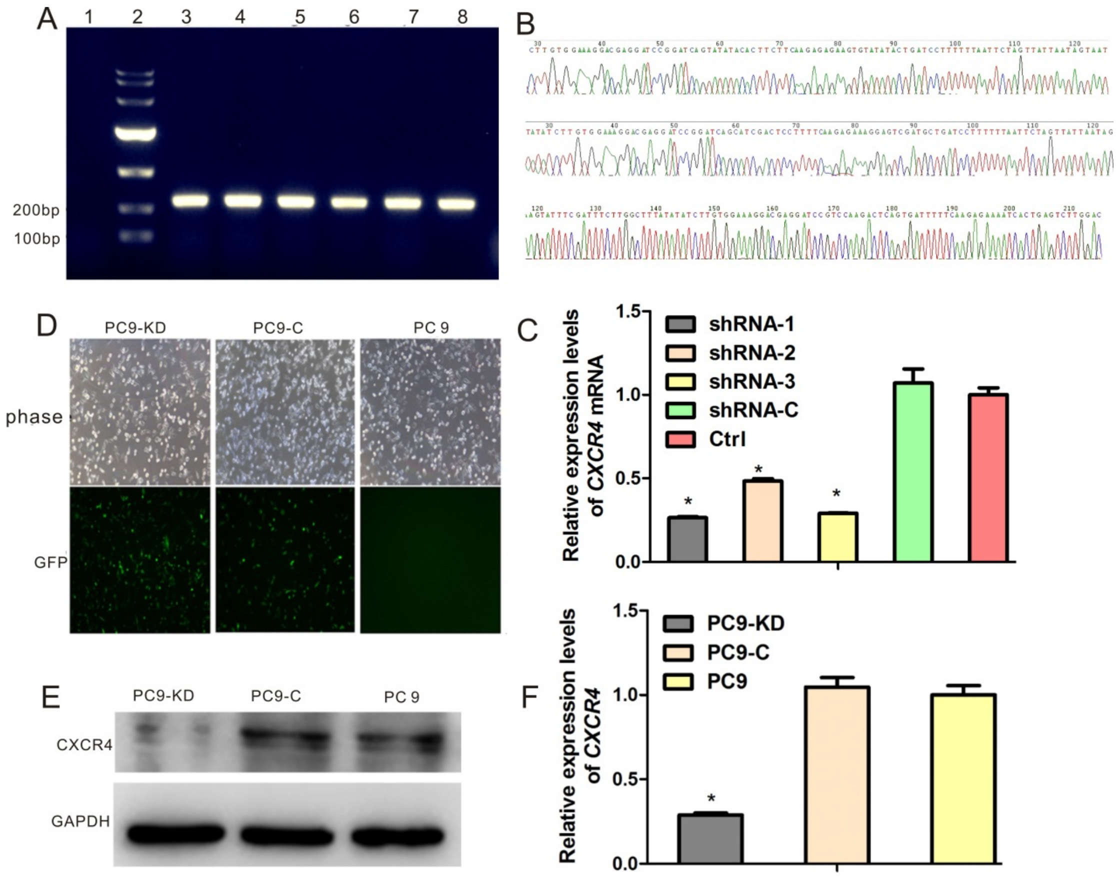

A
1 2 3 4 5 6 7 8
200bp
100bp
B
D
PC9-KD
PC9-C
PC 9
phase
GFP
C
Relative expression levels of CXCR4 mRNA
shRNA-1
shRNA-2
shRNA-3
shRNA-C
Ctrl
1.5
1.0
0.5
0.0
*
E
CXCR4
GAPDH
F
Relative expression levels of CXCR4
PC9-KD
PC9-C
PC9

Figure S3

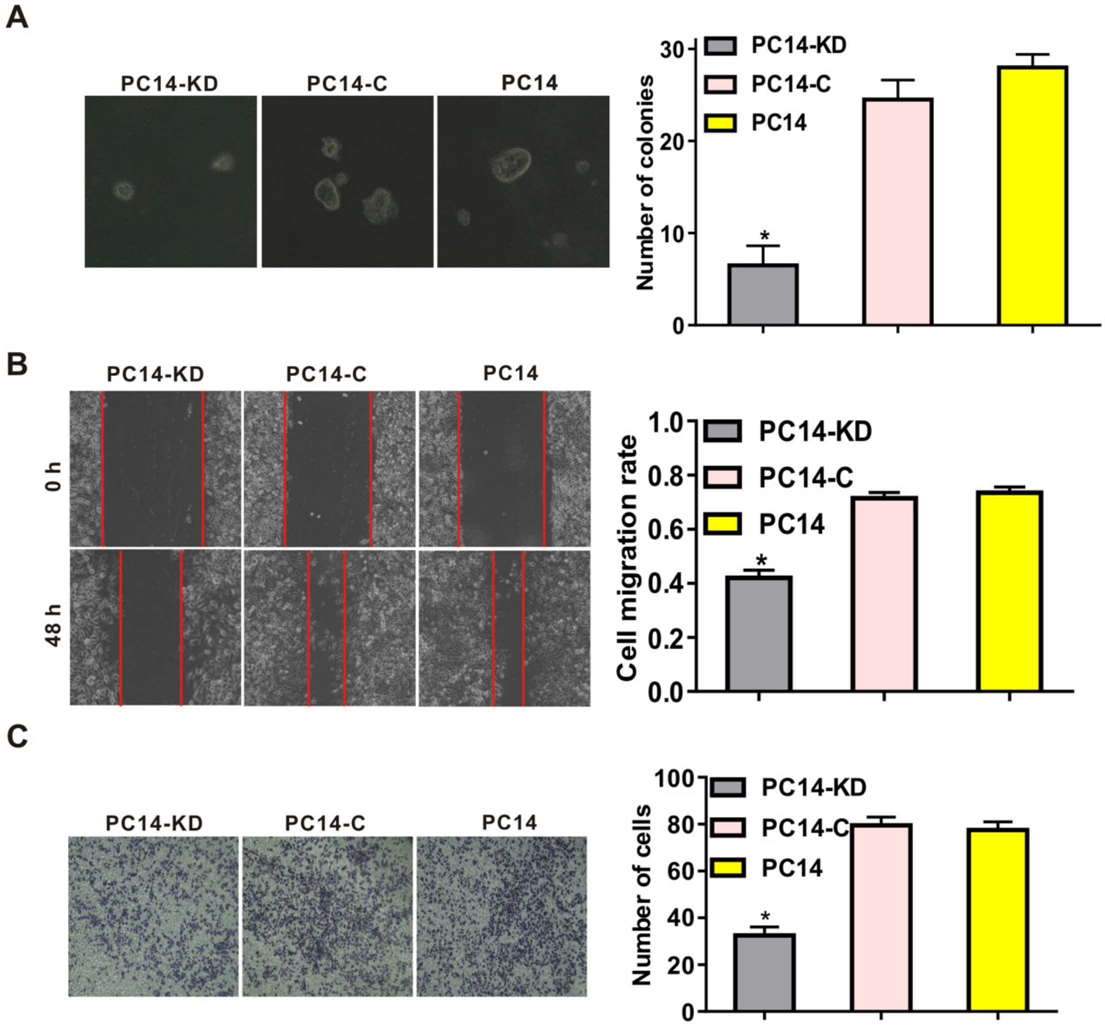
A
PC14-KD
PC14-C
PC14
Number of colonies
30
20
10
0
*
B
0 h
48 h
Cell migration rate
1.0
0.8
0.6
0.4
0.2
0.0
C
Number of cells
100
80
60
40
20
0

Table S1. ShRNA sequences for targeting *CXCR4*

Primer name	Orientation	Sequence (5'→3')
shRNA1	Forward	gatccGGATCAGCATCGACTCCTTTTCAAGAGAAAGGAGTCGATGCTGATCCTTTTTTg
	Reverse	aattcAAAAAAGGATCAGCATCGACTCCTTTCTCTTGAAAAGGAGTCGATGCTGATCCg
shRNA2	Forward	gatccGGATCAGTATATACACTTCTTCAAGAGAGAAGTGTATATACTGATCCTTTTTTg
	Reverse	aattcAAAAAAGGATCAGTATATACACTTCTCTCTTGAAGAAGTGTATATACTGATCCg
shRNA3	Forward	gatccGCAAGGCAGTCCATGTCATTTCAAGAGAATGACATGGACTGCCTTGCTTTTTTg
	Reverse	aattcAAAAAAGCAAGGCAGTCCATGTCATTCTCTTGAAATGACATGGACTGCCTTGCg
shRNAc	Forward	ccggtGCTTCGACATTTAACCAATTTCAAGAGAATTGGTTAAATGTCGAAGCTTTTTTg

Table S2. Sequences of the primers used in qRT-PCR assays.

Gene		Sequence (5’→3’)
CXCR4	Forward	GGAGAGTTGTAGGATTCTAC
	Reverse	CCTCGGTGTAGTTATCTGAAG
GAPDH	Forward	GGCGATGCTGGCGCTGAGTAC
	Reverse	GAGGCTGTTGTCATACTTCTC
hsa_circRNA_0056616	Forward	TTTTGCCTCAGAGCATACCT
	Reverse	GTCTTTGTTCTTTACTTCTCCCA

Table S3. CXCR4-related circRNAs identified in database search

CircRNA ID	Genomic length (bp)	Spliced length (bp)	Scores	Repeats	Annotation
has_circ_0056615	3389	3389	NA	NA	ALT_ACCEPTOR.CDS.coding, OVCOED, OVEXON, upstream_start, UTR3, UTR5
has_circ_0056616	3807	1674	NA	NA	ANNOTATED.CDS.coding, OVCOED, OVEXON, UTR3, UTR5
has_circ_0117403	253	253	3	NA	ALT_ACCEPTOR.ALT_DONOR., CDS.coding, INTERNAL, OVCOED, OVEXON, UTR3

NA: not available
